# Multicenter Experience with Nonischemic Multiport Laparoscopic and Laparoendoscopic Single-Site Partial Nephrectomy Utilizing Bipolar Radiofrequency Ablation Coagulator

**DOI:** 10.1155/2011/636537

**Published:** 2011-06-20

**Authors:** Wassim M. Bazzi, Mohamad E. Allaf, Jared Berkowitz, Hany N. Atalah, Sijo Parekattil, Ithaar H. Derweesh

**Affiliations:** ^1^Division of Urology, Department of Surgery, University of California San Diego School of Medicine, San Diego, CA 92103, USA; ^2^James Buchanan Brady Urological Institute, Johns Hopkins University School of Medicine, Baltimore, MD 21287, USA; ^3^Department of Urology, University of Florida College of Medicine, Gainesville, FL 32610, USA; ^4^Division of Urologic Oncology, Moores UCSD Cancer Center, 3855 Health Sciences Drive, 0987 La Jolla, CA 92093, USA

## Abstract

*Objective*. To investigate feasibility of multiport and laparoendoscopic single-site (LESS) nonischemic laparoscopic partial nephrectomy (NI-LPN) utilizing bipolar radiofrequency coagulator. *Methods*. Multicenter retrospective review of 60 patients (46 multiport/14 LESS) undergoing NI-LPN between 4/2006 and 9/2009. Multiport and LESS NI-LPN utilized Habib 4X bipolar radiofrequency coagulator to form a hemostatic zone followed by nonischemic tumor excision and renorrhaphy. Demographics, tumor/perioperative characteristics, and outcomes were analyzed. *Results*. 59/60 (98.3%) successfully underwent NI-LPN. Mean tumor size was 2.35 cm. Mean operative time was 160.0 minutes. Mean estimated blood loss was 131.4 mL. Preoperative/postoperative creatinine (mg/dL) was 1.02/1.07 (*P* = .471). All had negative margins. 12 (20%) patients developed complications. 3 (5%) developed urine leaks. No differences between multiport and LESS-PN were noted as regards demographics, tumor size, outcomes, and complications. *Conclusion*. Initial experience demonstrates that nonischemic multiport and LESS-PN is safe and efficacious, with excellent short-term preservation of renal function. Long-term data are needed to confirm oncological efficacy.

## 1. Introduction

In 2009, there were approximately 57,760 new cases of kidney cancer with 12,980 deaths in the United States [1]. Increasing incidence of small renal masses (SRMs) [2], along with an increased awareness of the metabolic consequences of removal of normally functioning renal tissue by radical nephrectomy [3–7], has led to a paradigm shift of surgical management with greater emphasis on nephron sparing and minimally invasive approaches [8, 9]. While laparoscopic partial nephrectomy (LPN) is becoming recognized as a standard for management of SRM, concerns regarding prolonged warm ischemia times continue to forestall its widespread adoption [8, 9]. 

By combining working trocar sites and the eventual extraction site into a single location, laparoendoscopic single-site (LESS) surgery further limits the invasiveness of laparoscopy and may enhance advantages associated with traditional laparoscopy. Reduced incisional morbidity and improved cosmesis have largely sparked a growing interest in the utilization of this technique to perform upper tract urologic surgery [10–13]. Recent reports have demonstrated the feasibility of LESS partial nephrectomy [12, 13]. 

The Habib 4X (Angiodynamics, Queensbury, NY) is a bipolar radiofrequency coagulation device which has been used for nonischemic resections of the liver [14] as well as kidney [15]. We investigated the feasibility, efficacy, and renal functional outcomes of multiport and LESS nonischemic LPN utilizing the laparoscopic Habib 4X.

## 2. Patients and Methods

Multicenter retrospective review of 60 patients (31M/29F, mean age of 57.9 years) who underwent nonischemic (NI-) LPN between 4/2006 and 9/2009. Mean followup was 16.3 ± 10.5 months. Institutions (performing surgeons) were as follows: University of California San Diego (IHD), Johns Hopkins University (MEA), and University of Florida (SP). Selection criteria were patients with contrast-enhancing small renal masses that would be technically amenable for multiport or LESS partial nephrectomy (PN).

### 2.1. Surgical Technique Multiport LPN

**Figure 1:**
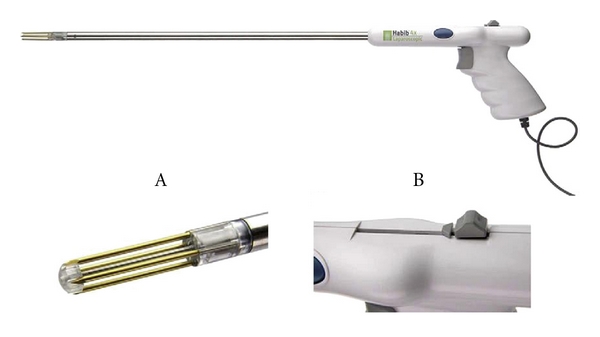
Laparoscopic Habib 4X (Angiodynamics, Queensbury, NY).

**Figure 2:**
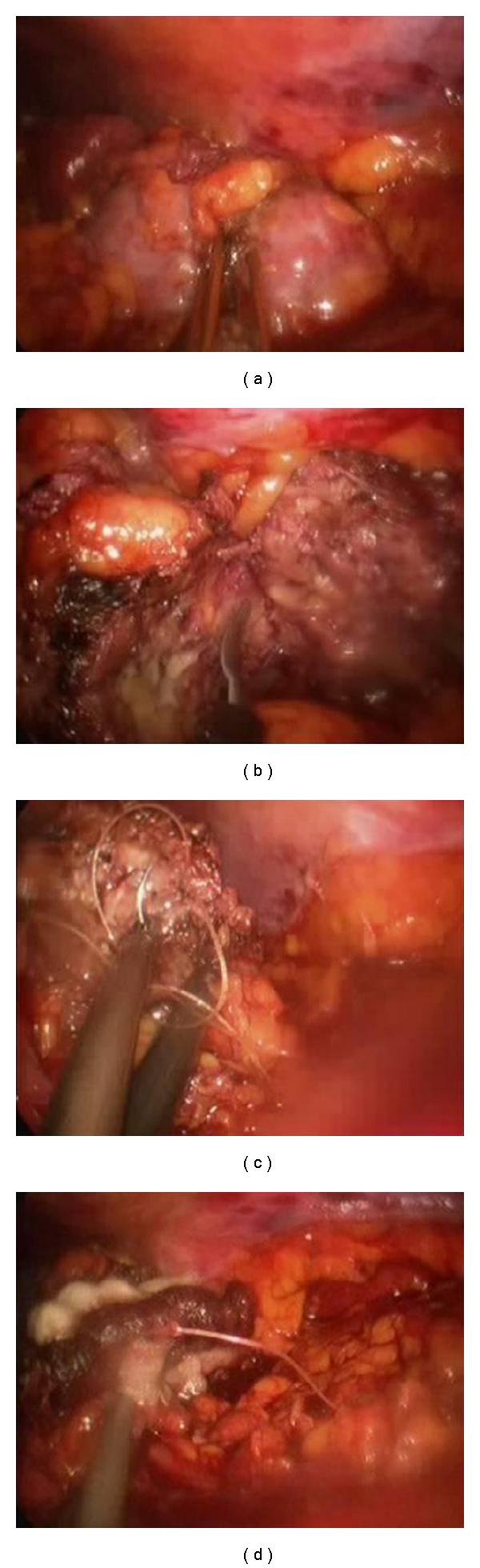
(a) Laparoscopic Habib 4X circumscribing left kidney lower pole tumor; (b) nonischemic cold excision of renal tumor; (c) sutured renorrhaphy; and (d) FloSeal application for hemostasis.

All procedures were performed by transperitoneal laparoscopic or retroperitoneoscopic approaches [16]. Briefly, after obtaining transperitoneal or retroperitoneoscopic access, the kidney was mobilized following ureteral identification. Hilar control was obtained, and the vessels were prepared for possible ischemic occlusion if the need arose. The kidney was then defatted to expose the tumor or region of the tumor. The tumor was circumscribed with at least a 1.0 cm margin utilizing the Habib 4X (Figure 1) to create a zone of hemostasis (Figure 2(a)). The Habib 4X utilizes the RITA 1500X generator which operates at 480 KHz with a 125 W power that has 4 tines (3 cm in length and 1 cm apart) with 2 planes of coagulation created by each 2 tines. The depth of coagulation is controlled by the depth of insertion, and the duration of coagulation is controlled by the surgeon with on-demand repeat. The duration is gradually decreased because of the rise in tissue impedance from charring. We typically use a 50 W setting but that is usually dropped to 25 W for larger vessels to allow for longer duration of coagulation. Parenchymal bleeding is encountered when the tines are removed and that is controlled by reinsertion over the site of bleeding. 

**Figure 3:**
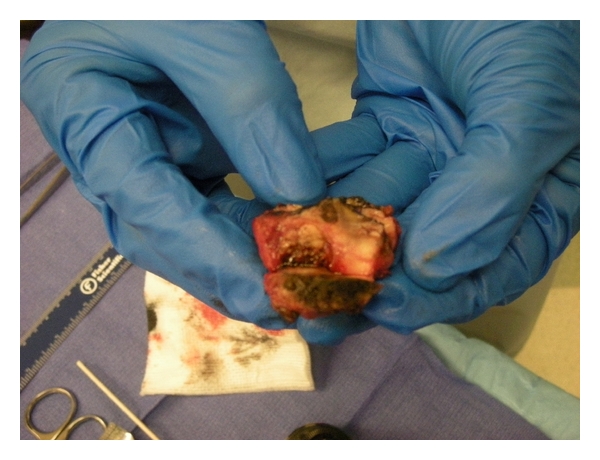
Resected left lower pole inked margin demonstrated by intraoperative pathology consult.

After the tumor is clearly demarcated by a zone of coagulation, it is excised by cold EndoShears (Covidien, Mansfield, Mass) (Figure 2(b)). Standard sutured renorrhaphy (Figure 2(c)) was then performed with sutured collecting system closure, and FloSeal (Baxter, Deerfield, Ill) was utilized as a hemostatic adjunct, and the parenchymal defect was closed over an oxidized cellulose mesh bolster with interrupted sutures using LapraTy absorbable clips (Ethicon, Cincinnati, Ohaio) [17] (Figure 2(d)). Specimens were intraoperatively evaluated by a pathologist for margin status (Figure 3). A Jackson-Pratt drain was placed to monitor for delayed bleed or leaks.

### 2.2. Surgical Technique for Nonischemic LESS-PN

**Figure 4:**
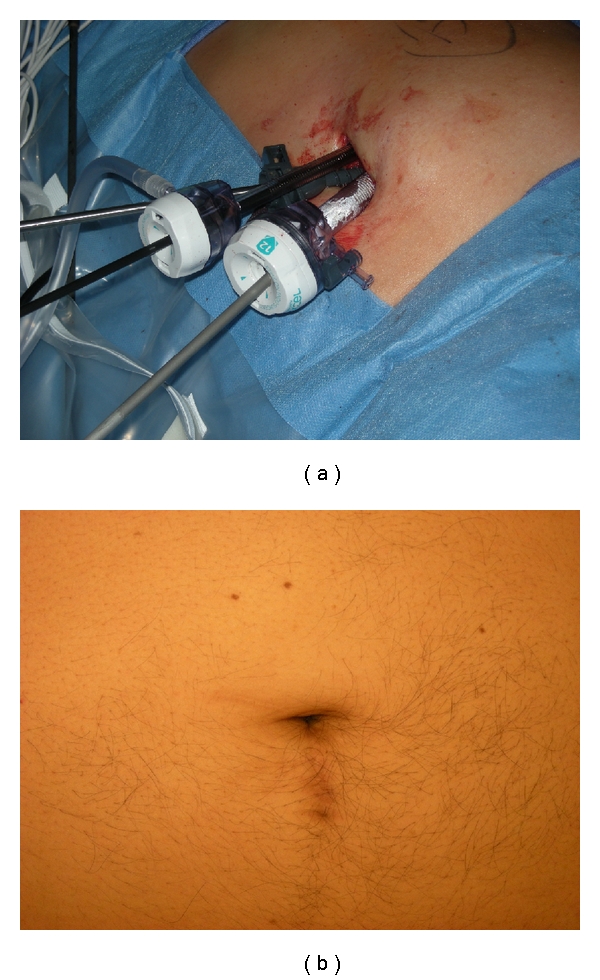
(a) LESS platform, periumbilical incision for left partial nephrectomy, demonstrating in cranial to caudal direction (left to right): 5 mm Extra-long Xcel trocar, 5 mm short nonshielded trocar, and 12 mm Xcel trocar (with Habib 4X deployed through the 12 mm Trocar); (b) periumbilical incision 6-month status after LESS-PN.

After general anesthesia, the patient is placed in a modified flank position (with the patient at a 30-degree angle with the kidney rest up and the table flexed). A 3-4 cm periumbilical incision is made and carried down to the anterior abdominal wall fascia. A 5 mm extra long (150 mm length) Xcel trocar (Ethicon-Endosurgery, Cincinnati, Ohaio) is then inserted at the most cranial aspect of this incision, at the junction of the umbilicus with the fascia; pneumoperitoneum to 15 mmHg is obtained, and a 5 mm zero-degree 35 cm long laparoscope (Strkyer, Kalamazoo, Mich) is inserted to inspect the abdomen; subsequently, a 65 mm long, nonshielded low profile (65 mm length) trocar (Ethicon) is inserted under direct vision at a position of 1.0–1.5 cm caudad to the initial port, followed by the insertion of a standard length (100 mm) 12 mm Xcel trocar (Ethicon) at the most caudal aspect of the incision; another 1.0–1.5 cm caudad to the prior low profile port. We minimized the intracorporeal profile of the Xcel trocars, and that in conjunction with the variety of trocar lengths allowed us to stagger the external profiles in order to minimize instrument clashing (Figure 4). 

Tissue dissection is largely performed with standard extra long laparoscopic instruments (nonlocking laparoscopic deBakey bowel forceps, right angle dissector, Maryland dissector, endoshears) and 5 mm harmonic ACE 36 cm curved shears (Ethicon). Utilization of extra-long instruments creates extracorporeal triangulation which compensates for the intracorporeal triangulation afforded by spaced trocars in multisite laparoscopy. Following the takedown of the white line of Toldt, the 0-degree laparoscope is exchanged for a 5 mm, 45 cm, 30-degree laparoscope with a right angle adaptor and inline camera head (Strkyer). 

Initial surgical steps including colonic mobilization, ureteral identification, and vascular dissection are identical to multiport LPN. The standard techniques of LPN are recapitulated with few modifications [16]. The Habib 4X then was utilized to achieve a zone of parenchymal hemostasis in the absence of ischemic renal conditions prior to cold tumor excision and renorrhaphy, as described above [15–17].

### 2.3. Postoperative Protocol

It consisted of first night bed rest, then rapid mobilization, and advancement of diet as tolerated with monitoring of renal function, urine, and drain outputs. If no evidence of a leak was noted, the drain and foley were discontinued prior to discharge. Outpatient followup consisted of serial physical exams, serum chemistries and hematology labs, and radiographic surveillance.

### 2.4. Data Analysis

We analyzed patient demographics (age, gender, BMI, and race), operative outcomes (operative time, estimated blood loss, and collecting system entry), pathologic outcomes (size, margin status, and final pathology), and complications (urologic and nonurologic). TNM stage was defined by the American Joint Committee on Cancer 2002 [18]. Renal function was determined by serum creatinine (mg/dL) measurement and calculated estimated glomerular filtration rate (eGFR) using the MDRD formula (eGFR (in mL per minute per 1.73 m^2^) = 186 × sCr − 1.154 × age^0.203^ × (0.742 if female) × (1.210 if black)) [19]. Means were compared between the two groups (multiport and LESS) using *t*-tests/ANOVA and fisher's exact test for continuous and categorical variables, respectively. All reported *P* values were based on two-sided tests of significance, with *P* < .05 considered to indicate statistical significance.

## 3. Results

59/60 (98.3%) patients successfully underwent nonischemic LPN and LESS-PN. One patient, an attempted LESS-PN on a patient with a posterior left upper pole mass, was converted to open nonischemic PN due to dense upper pole adhesions and nonprogression due to a prior history of multiple intra-abdominal procedures and radiation. There were no conversions to ischemic partial nephrectomy, open surgery, or radical nephrectomy. 

Table 1 demonstrates demographics and tumor characteristics. Mean followup was 16.3 ± 10.5 months and was significantly longer for multiport LPN (18.8 ± 10.8) than for LESS-PN (9.4 ± 2.6, *P* = .004). Mean age was 57.9 ± 12.7 years. 31 (51.7%) were male, and 29 (48.3%) were female. Mean BMI was 28.4 ± 0.5 kg/m^2^. 66.7% (40) were Caucasian and 33.3% (20) were other. Mean tumor size was 2.35 ± 1.30 cm. No significant difference was found between MPL and LESS-PN groups with respect to age, gender/race distribution, BMI, or tumor size. 

Table 2 shows perioperative parameters, outcomes, and complications. Mean operative time was 164.0 ± 48.8 minutes, and mean estimated blood loss was 131.4 ± 98.1 mL. Collecting system entry was made in 17/60 (28.3%). Mean hospital length of stay was 2.60 ± 1.17 days. Final pathology was renal cell carcinoma in 34 (56.7%) and Benign histology in 26 (43.3%) (oncocytoma (5), AML (13), other (8)). All had negative surgical margins. Preoperative serum creatinine (mg/dL) and creatinine at the time of last followup were 1.02 ± 0.41 and 1.07 ± 0.45 (*P* = .471). Preoperative eGFR (mL/min/1.73 m^2^) and eGFR at the time of the last followup were 81.4 ± 26.9 and 77.7 ± 29.6 (*P* = .471). There were no significant differences between multiport and LESS-PN with respect to mean operative time, EBL, collecting system entry, length of stay, preoperative/postoperative creatinine and eGFR, and pathological distribution. At the last followup, all patients were alive and disease-free. 

There were 12 complications in 12 patients (20%) in our series. 1 (1.7%) patient developed clot obstruction which required stent placement on postoperative day 2. 3 (5%) patients developed urine leaks which resolved with conservative management (two were early leaks managed with Jackson-Pratt drain continuing for 1 and 2 weeks, respectively. 1 patient developed a delayed urinary leak treated with percutaneous drain placement). All leaks were resolved with conservative management. 3 patients (5%) developed postoperative ileus managed conservatively. 1 patient (1.7%) developed each of the following: atrial fibrillation, cystitis, diverticulitis, pneumonia, and pneumothorax. 1 patient received a platelet and blood transfusion, who had a history of preoperative anemia and idiopathic thrombocytopenic purpura. There were no significant differences between the groups with respect to complication and leak rates.

## 4. Discussion

By duplicating principles of open partial nephrectomy, laparoscopic partial nephrectomy has demonstrated equivalence in oncological outcomes [8, 9, 23] while providing the benefits of minimally invasive surgery such as improved cosmesis, lower narcotic requirements, shorter hospital stays, and more rapid return to normal activities [24, 25]. Adoption of laparoscopic partial nephrectomy, however, continues to be hampered by concerns regarding the achievement of reliable hemostasis [26] and prolonged warm ischemia time and its possible consequences as regards long-term renal function [8, 9, 27, 28]. 

Interest in reducing or eliminating ischemic renal occlusion has spurred the development of a variety of strategies including sutureless renorrhaphy [29, 30], superselective embolization of segmental vessels [31], and energy-based nonischemic renal resection utilizing laser [32, 33], microwave tissue ablation [34], and monopolar radiofrequency devices [35, 36]. Partial nephrectomy utilizing bipolar radiofrequency device was first investigated by Pareek et al. [37] in a porcine model. In humans, Andonian et al. [38] were the first to report success in humans with renal masses smaller than 2 cm utilizing the Habib 4X. White et al. [15] reported a randomized controlled trial utilizing the Habib 4X in open partial nephrectomy versus ischemic open partial nephrectomy, reporting a significant decrease in blood loss and operative time for tumors with mean size of 3.3 cm. Zeltser et al. [39] used the laparoscopic Habib 4X in a porcine model with good results, and recently, Nadler et al. [20] reported their clinical experience in 16 patients who underwent a hybrid laparoscopic and robotic-assisted laparoscopic partial nephrectomy with the Habib 4X. They noted a median blood loss of 125 mL, median operative time of 435 minutes, mean hospital stay of 2.6 days, and with good renal functional preservation (preoperative/postoperative serum creatinine of 1.03/1.10). Importantly, all patients had negative margins, and none were converted to ischemic or open technique. Our multicenter experience, utilizing pure laparoscopy, demonstrates similar outcomes with respect to estimated blood loss (131 mL), hospital stay (2.60 days), and preservation of renal function (preoperative/postoperative serum creatinine 1.02/1.07, Table 2) to those achieved by Nadler et al. [20] with decreased operative time (164 minutes) and with the cost savings of using a purely laparoscopic platform [40].

Warm ischemia time is a major road block in performing laparoscopic partial nephrectomy [27]. Indeed, while refinement of technique and emerging reports on the feasibility of robotically assisted laparoscopy for nephron sparing surgery, concern continues regarding potentially longer warm ischemia times for minimally invasive surgery. On the other hand, concerns have been raised regarding energy-based modalities and potential limitations with respect to the assessment of margins status as well as efficacy of collecting system closure and reconstruction. By eliminating warm ischemia and facilitating complex reconstruction with robotically assisted laparoscopy, Nadler et al. [20] have pointed the way in expanding the range of utilization of nephron sparing surgery. Indeed, our experience with 17/60 (28.3% of patients/tumors, Table 2) collecting system entries and sutured renorrhaphy (Figure 2) confirms that the utilization of nonischemic technique is ideally suited for a minimally invasive surgery as elimination of ischemia time will not only allay concerns about the prolongation of ischemia time and its deleterious effects, but also permit complex reconstruction and therefore ultimately enhance the adoption of minimally invasive nephron sparing surgery for a wider variety of tumors. Our urinary leak rate of 5% (3 patients), all of whom resolved with conservative measures, is consistent with rates reported in large series of laparoscopic partial nephrectomy [8, 20–22]. Furthermore, as demonstrated by our results, adhering to a 1 cm margin outside the tumor permitted the Habib 4X, a four-pronged device (prongs are set 8 mm apart) with minimal lateral coagulation spread, to achieve a zone of coagulation that was away from the tumor. Indeed, our 0% negative pathologic margins bear this out (Table 2). 

Indeed, in addition to a comparable leak rate, our outcomes as regards to blood loss, operative time, margin positivity, and renal functional preservation compare very favorably with large published multicenter studies (Table 3) [8, 20–22]. These data demonstrate that nonischemic laparoscopic partial nephrectomy utilizing the Habib 4X is not only feasible but has a similar safety profile to established techniques and does not compromise oncological outcomes with respect to margin positivity.

LESS-PN allows for the extraction of the enhancing renal lesion, a definitive histologic confirmation with excellent preservation of renal function in this series. In a recent publication, Kaouk and Goel utilized a nonischemic technique to perform LESS-PN. After PN, these authors achieved hemostasis using ABC, Surgicel and a variety of surgical adhesives; however, due to inability to achieve adequate hemostasis in one case, they had to convert to multiport laparoscopy [12]. Our experience with 14 LESS-PN not only confirms the feasibility of this procedure, but in a well-matched cohort (Table 1) demonstrates similar outcomes (Table 2) to our multiport LPN group. Indeed, the Habib 4X, which easily fits through the 12 mm laparoscopic port, was a key facilitator of the LESS-PN approach, by allowing controlled excision of the mass through a precreated hemostatic plane around the tumor, and therefore minimizing instrument clashing and potential loss of hemostatic control in a crucial portion of the case. Furthermore, by creating a hemostatic field, we were able to proceed with the renorrhaphy in a meticulous manner and be able to surmount the limitations of the LESS platform (instrument clashing, triangulation) in an environment without the pressure of ischemic time. While further investigation and comparison is necessary, this preliminary series demonstrates that LESS-PN is safe and technically feasible method for performing complex renal surgery while maintaining strict adherence to oncologic principles. Our encouraging results are the first reported comparison between multiport and LESS-PN. Further studies, in addition to a comparison of quality-of-life outcomes, are necessary to delineate what, if any specific advantages, may lie with the LESS approach.

Our data is limited by its retrospective nature, limited numbers, and short-term follow-up. Nonetheless, we are encouraged by the preliminary findings of our experience. Further prospective comparison with ischemic partial nephrectomy with longer-term outcomes is requisite.

## 5. Conclusion

Initial experience demonstrates that nonischemic L-NSS utilizing Habib 4X is safe and efficacious, with excellent short-term preservation of renal function. In addition to longer-term followup, direct prospective comparison to ischemic NSS is requisite to confirm the renal functional preservation and oncological efficacy of this technique.

## Figures and Tables

**Table 1:** Patient demographics and tumor characteristics.

	Total *N* = 60	Multiport LPN *N* = 46	LESS-PN *N* = 14	*P* value
Mean f/u (months)	16.3 ± 10.5	18.8 ± 10.8	9.4 ± 2.6	.004
Mean age (yrs.)	57.9 ± 12.7	57.4 ± 13.3	59.3 ± 11.7	.627
Gender				.547
Male	31 (51.7%)	25 (54.3%)	6 (42.9%)	
Female	29 (48.3%)	21 (46.7%)	8 (57.1%)	
Mean BMI (Kg/m^2^)	28.4 ± 0.5	28.0 ± 4.8	28.7 ± 5.5	.737
Race/Ethnicity				.195
Caucasian	40 (66.7%)	33 (71.6%)	7 (50%)	
Other	20 (33.3%)	13 (28.3%)	7 (50%)	
Laterality				.770
Right	28 (46.7%)	22 (47.8%)	6 (42.9%)	
Left	32 (52.3%)	24 (52.2%)	8 (57.1%)	
Tumor location				.043
Upper pole	13 (21.7%)	10 (21.8%)	3 (21.4%)	
Mid-pole	20 (33.3%)	14 (30.4%)	6 (42.9%)	
Lower pole	27 (45.0%)	22 (47.8%)	5 (35.7%)	
Tumor location				.241
Anterior	34 (56.7%)	25 (54.3%)	9 (64.3%)	
Posterior	25 (41.7%)	20 (43.5%)	5 (35.7%)	
Central	1 (1.6%)	1 (2.2%)	0 (0%)	
Tumor size (cm)	2.35 ± 1.30	2.47 ± 1.38	1.95 ± 0.92	.189

**Table 2:** Perioperative parameters, outcomes, and complications.

	Total *N* = 60	Multiport LPN *N* = 46	LESS-PN *N* = 14	*P* value
Mean OR time (min)	164.0 ± 48.8	159.9 ± 52.1	177.4 ± 34.0	.242
Mean EBL (mL)	131.4 ± 98.1	126.6 ± 94.7	148.1 ± 112	.491
Collecting system entry	17 (28.3%)	11 (23.9%)	6 (42.9%)	.190
LOS (days)	2.60 ± 1.17	2.61 ± 1.23	2.57 ± 0.94	.917
Preoperative serum creatinine (mg/dL)	1.02 ± 0.41	1.06 ± 0.43	0.89 ± 0.33	.187
Postoperative serum creatinine (mg/dL)	1.07 ± 0.45	1.10 ± 0.47	0.97 ± 0.35	.340
Preoperative eGFR (mL/min/1.73 m^2^)	81.4 ± 26.9	79.4 ± 27.2	88.1 ± 26.0	.293
Postoperative eGFR (mL/min/1.73 m^2^)	77.7 ± 29.6	77.4 ± 31.0	78.5 ± 25.1	.905
Final pathology				.759
RCC	34 (56.7%)	27 (58.7%)	7 (50%)	
Benign histology	26 (43.3%)	19 (41.3%)	7 (50%)	
Negative margins	60 (100%)	46 (100%)	14 (100%)	1.000
Complications	12 (20%)	9 (10.5%)	3 (21.4%)	1.000
Urine leak	3 (5%)	2 (4.3%)	1 (7.1%)	.556

**Table 3:** Comparison of our data to reported world literature.

	Our series	Gill et al. [8]	Nadler et al. [20]	Celia et al. [21]	Crepel et al. [22]
Number of patients	60	771	16	592	91
Mean age (yrs)	57.9	59.4	—	—	—
Tumor size (cm)	2.35	2.7	—	2.2	2.7
Hospital days	2.6	3.3	2.6	—	9.1
Operative time (min)	164.2	300	435	—	163
Estimated blood loss (mL)	131.4	201	125	—	363
Urine leak rate	5.0%	2.3%	0.0%	2.1%	12.1%
Positive margins	0%		0%	2%	
Pre/Post op creatinine (mg/dL)	1.02/1.07	1.01/1.18	1.03/1.10	—	—

## References

[1] Jemal A, Siegel R, Ward E, Hao Y, Xu J, Thun MJ (2009). Cancer statistics, 2009. *CA: Cancer Journal for Clinicians*.

[2] Kane CJ, Mallin K, Ritchey J, Cooperberg MR, Carroll PR (2008). Renal cell cancer stage migration: Analysis of the National Cancer Data Base. *Cancer*.

[3] Thompson RH, Boorjian SA, Lohse CM (2008). Radical nephrectomy for pT1a renal masses may be associated with decreased overall survival compared with partial nephrectomy. *The Journal of Urology*.

[4] Huang WC, Levey AS, Serio AM (2006). Chronic Renal Insufficiency after nephrectomy in patients with renal cortical tumours: a retrospective cohort study. *Lancet Oncology*.

[5] Go AS, Chertow GM, Fan D, McCulloch CE, Hsu CY (2004). Chronic renal insufficiency and the risks of death, cardiovascular events, and hospitalization. *The New England Journal of Medicine*.

[6] Lucas SM, Stern JM, Adibi M, Zeltser IS, Cadeddu JA, Raj GV (2008). Renal function outcomes in patients treated for renal masses smaller than 4 cm by ablative and extirpative techniques. *Journal of Urology*.

[7] Malcolm JB, Bagrodia A, Derweesh IH (2009). Comparison of rates and risk factors for development of chronic renal insufficiency, proteinuria, and metabolic acidosis following radical or partial nephrectomy. *British Journal of Urology International*.

[8] Gill IS, Kavoussi LR, Lane BR (2007). Comparison of 1,800 laparoscopic and open partial nephrectomies for single renal tumors. *Journal of Urology*.

[9] Campbell SC, Novick AC, Belldegrun A (2009). Guideline for management of the clinical T1 renal mass. *Journal of Urology*.

[10] Raman JD, Cadeddu JA, Rao P, Rane A (2008). Single-incision laparoscopic surgery: initial urological experience and comparison with natural-orifice transluminal endoscopic surgery. *British Journal of Urology International*.

[11] Ponsky LE, Cherullo EE, Sawyer M, Hartke D (2008). Single access site laparoscopic radical nephrectomy: initial clinical experience. *Journal of Endourology*.

[12] Kaouk JH, Goel RK (2009). Single-port laparoscopic and robotic partial nephrectomy. *European Urology*.

[13] Aron M, Canes D, Desai MM, Haber GP, Kaouk JH, Gill IS (2009). Transumbilical single-port laparoscopic partial nephrectomy. *British Journal of Urology International*.

[14] Pai M, Jiao LR, Khorsandi S, Canelo R, Spalding DRC, Habib NA (2008). Liver resection with bipolar radiofrequency device. *Hepatobiliary*.

[15] White WM, Klein FA, Waters WB (2008). Nephron sparing surgery using a bipolar radio frequency resection device. *Journal of Urology*.

[16] Gill IS, Desai MM, Kaouk JH (2002). Laparoscopic partial nephrectomy for renal tumor: duplicating open surgical techniques. *Journal of Urology*.

[17] Orvieto MA, Chien GW, Laven B, Rapp DE, Sokoloff MH, Shalhav AL (2004). Eliminating knot tying during warm ischemia time for laparoscopic partial nephrectomy. *Journal of Urology*.

[18] AJCC (2002). *Cancer Staging Manual*.

[19] Levey AS, Bosch JP, Lewis JB, Greene T, Rogers N, Roth D (1999). A more accurate method to estimate glomerular filtration rate from serum creatinine: a new prediction equation. *Annals of Internal Medicine*.

[20] Nadler RB, Perry KT, Smith ND (2009). Hybrid laparoscopic and robotic ultrasound-guided radiofrequency ablation-assisted clampless partial nephrectomy. *Urology*.

[21] Celia A, Zeccolini G, Guazzoni G (2008). Laparoscopic nephron sparing surgery: a multi-institutional European survey of 592 cases. *Archivio Italiano di Urologia e Andrologia*.

[22] Crepel M, Bernhard JC, Bellec L (2007). Comparison of open and laparoscopic partial nephrectomy: a french multicentre experienceComparaison de la néphrectomie partielle par voie laparoscopique et par voie ouverte: une expérience multicentrique française. *Progres en Urologie*.

[23] Allaf ME, Bhayani SB, Rogers C (2004). Laparoscopic partial nephrectomy: evaluation of long-term oncological outcome. *Journal of Urology*.

[24] Klingler HC, Remzi M, Janetschek G, Marberger M (2003). Benefits of laparoscopic renal surgery are more pronounced in patients with a high body mass index. *European Urology*.

[25] Burgess NA, Koo BC, Calvert RC, Hindmarsh A, Donaldson PJ, Rhodes M (2007). Randomized trial of laparoscopic *ν* open nephrectomy. *Journal of Endourology*.

[26] Walters RC, Collins MM, L’Esperance JO (2006). Hemostatic techniques during laparoscopic partial nephrectomy. *Current Opinion in Urology*.

[27] Lane BR, Babineau DC, Poggio ED (2008). Factors predicting renal functional outcome after partial nephrectomy. *Journal of Urology*.

[28] Thompson RH, Frank I, Lohse CM (2007). The impact of ischemia time during open nephron sparing surgery on solitary kidneys: a Multi-Institutional Study. *Journal of Urology*.

[29] Johnston WK, Montgomery JS, Seifman BD, Hollenbeck BK, Wolf JS (2005). Fibrin glue V sutured bolster: lessons learned during 100 laparoscopic partial nephrectomies. *Journal of Urology*.

[30] Derweesh IH, Malcolm JB, Diblasio CJ, Mehrazin R, Jackson S (2008). Sutureless laparoscopic heminephrectomy: safety and efficacy in physiologic and chronically obstructed porcine kidney. *Surgical Innovation*.

[31] Gallucci M, Guaglianone S, Carpanese L (2007). Superselective embolization as first step of laparoscopic partial nephrectomy. *Urology*.

[32] Moinzadeh A, Gill IS, Rubenstein M (2005). Potassium-titanyl-phosphate laser laparoscopic partial nephrectomy without hilar clamping in the survival calf model. *Journal of Urology*.

[33] Lotan Y, Gettman MT, Lindberg G (2004). Laparoscopic partial nephrectomy using holmium laser in a porcine model. *Journal of the Society of Laparoendoscopic Surgeons*.

[34] Terai A, Ito N, Yoshimura K (2004). Laparoscopic partial nephrectomy using microwave tissue coagulator for small renal tumors: usefulness and complications. *European Urology*.

[35] Tan YH, Young MD, L’Esperance JO, Preminger GM, Albala DM (2004). Hand-assisted laparoscopic partial nephrectomy without hilar vascular clamping using a saline-cooled, high-density monopolar radiofrequency device. *Journal of Endourology*.

[36] Herrell SD, Levin BM (2005). Laparoscopic partial nephrectomy: Use of the tissuelink*™* hemostatic dissection device. *Journal of Endourology*.

[37] Pareek G, Wilkinson ER, Schutt D (2005). Haemostatic partial nephrectomy using bipolar radiofrequency ablation. *British Journal of Urology International*.

[38] Andonian S, Adebayo A, Okeke Z, Lee BR (2008). Habib laparoscopic bipolar radiofrequency device: a novel way of creating an avascular resection margin in laparoscopic partial nephrectomy. *Journal of Laparoendoscopic and Advanced Surgical Techniques A*.

[39] Zeltser IS, Gupta A, Bensalah K (2008). Focal radiofrequency coagulation-assisted laparoscopic partial nephrectomy: a novel nonischemic technique. *Journal of Endourology*.

[40] Lotan Y, Cadeddu JA, Gettman MT (2004). The new economics of radical prostatectomy: cost comparison of open, laparoscopic and robot assisted techniques. *Journal of Urology*.

